# Evidence for Regulation of *ECM3* Expression by Methylation of Histone H3 Lysine 4 and Intergenic Transcription in *Saccharomyces cerevisiae*

**DOI:** 10.1534/g3.116.033118

**Published:** 2016-07-22

**Authors:** Elizabeth A. Raupach, Joseph A. Martens, Karen M. Arndt

**Affiliations:** Department of Biological Sciences, University of Pittsburgh, Pennsylvania 15260

**Keywords:** noncoding RNA, histone methylation, RNA polymerase II, cryptic unstable transcripts, Paf1 complex

## Abstract

Transcription of nonprotein-coding DNA is widespread in eukaryotes and plays important regulatory roles for many genes, including genes that are misregulated in cancer cells. Its pervasiveness presents the potential for a wealth of diverse regulatory roles for noncoding transcription. We previously showed that the act of transcribing noncoding DNA (ncDNA) across the promoter of the protein-coding *SER3* gene in *Saccharomyces cerevisiae* positions nucleosomes over the upstream activating sequences, leading to strong repression of *SER3* transcription. To explore the possibility of other regulatory roles for ncDNA transcription, we selected six candidate *S. cerevisiae* genes that express ncRNAs over their promoters and analyzed the regulation of one of these genes, *ECM3*, in detail. Because noncoding transcription can lead to changes in the local chromatin landscape that impinge on the expression of nearby coding genes, we surveyed the effects of various chromatin regulators on the expression of *ECM3*. These analyses identified roles for the Paf1 complex in positively regulating *ECM3* transcription through methylation of histone H3 at lysine 4 (K4) and for Paf1 in controlling the pattern of intergenic transcription at this locus. By deleting a putative promoter for the noncoding transcription unit that lies upstream of *ECM3*, we provide evidence for a positive correlation between intergenic transcription and *ECM3* expression. Our results are consistent with a model in which cotranscriptional methylation of histone H3 K4, mediated by the Paf1 complex and noncoding transcription, leads to activation of *ECM3* transcription.

Widespread pervasiveness of transcription is a common feature of eukaryotic genomes. The importance of pervasive nonprotein-coding transcription is highlighted by the results of the human genome project, which revealed that over 80% of the human genome displays biochemical activities associated with transcription in at least one cell type, even though only about 1% of the transcribed regions contain protein-coding exons (Venter *et al.* 2001; Borel *et al.* 2008; ENCODE Project Consortium 2012). Noncoding transcripts carry out a diverse array of regulatory functions. For example, microRNAs (miRNAs) associate with Argonaute proteins to regulate gene expression at a posttranscriptional level or by directing chromatin modifications (Bartel 2004). Several long noncoding RNAs (lncRNAs), such as *Xist* and *HOTAIR*, two important developmental regulators, associate with and direct the Polycomb repressive complex, PRC2, to specific genetic loci. PRC2 is then able to alter the local chromatin state and lead to the regulation of gene expression (Rinn *et al.* 2007; Lee 2009; Tsai *et al.* 2010; Simon and Kingston 2013). *PCGEM1*, a lncRNA associated with prostate cancer, directly binds the transcription factor c-Myc, activates transcription of c-Myc target genes, and regulates several metabolic pathways including nucleotide and lipid biosynthetic pathways and the tricarboxylic acid cycle (Hung *et al.* 2014).

In these examples, the noncoding RNA (ncRNA) molecule itself plays a regulatory role. The act of transcribing ncDNA can also alter the local chromatin environment and the regulation of neighboring genes. Transcription can alter the occupancy and positions of nucleosomes, posttranslational histone modifications, and potentially higher order chromatin structures. Our previous studies in *Saccharomyces cerevisiae* revealed an interesting role for the act of transcribing ncDNA, *SRG1*, at the promoter of a protein-coding gene, *SER3*, which represses transcription of *SER3* (Martens *et al.* 2004, 2005). The mechanism by which *SRG1* transcription represses *SER3* requires histone chaperones, Spt6 and the FACT complex, that travel with RNA polymerase II (Pol II) during transcription elongation. During transcription of *SRG1*, these histone chaperones place nucleosomes over the upstream regulatory sequences for the *SER3* gene, creating a barrier that prevents the transcriptional machinery from accessing the *SER3* promoter (Hainer *et al.* 2011).

Several other cases of gene regulation by ncDNA transcription have been reported. In *Schizosaccharomyces pombe*, the *fbp1+* gene is activated by stepwise displacement of nucleosomes over the *fbp1+* promoter, which is mediated by induction of a series of long noncoding transcripts across the promoter (Hirota *et al.* 2008). In maturing B cells, noncoding transcription at the IgL loci is required to evict histone H2A/H2B dimers to allow recombination factors to access the DNA for V(D)J recombination (Bevington and Boyes 2013). This example demonstrates that the regulatory potential for noncoding transcription is not limited to transcription and may be extended to all DNA-templated processes. In these examples, transcription of ncDNA exerts its regulatory effect by altering nucleosome occupancy.

At some loci, noncoding transcription has been shown to alter chromatin structure through posttranslational histone modifications. Transcribed loci display a characteristic set of posttranslational histone modifications, including acetylation of histones over promoter regions, trimethylation of H3 lysine 4 (H3 K4me3) at 5′ ends of transcription units, monoubiquitylation of H2B (at K123 in *S. cerevisiae* or K120 in humans) and methylation of H3 K79 throughout the bodies of transcription units, and methylation of H3 K36 at the 3′ ends of transcription units (reviewed in Smolle and Workman 2013). The histone modification states of nonprotein-coding transcribed regions can influence the expression of nearby protein-coding genes. For example, trimethylation of H3 K4 due to transcription of ncDNA at the *GAL1-10* locus is required for histone deacetylation, which leads to repression of *GAL1* and *GAL10* in the presence of glucose (Houseley *et al.* 2008; Pinskaya *et al.* 2009). Two other *S. cerevisiae* genes, *DCI1* and *DUR3*, are regulated by noncoding transcription across their promoters through a mechanism in which methylation of H3 K4 and subsequent Set3-mediated deacetylation of histones leads to gene repression (Kim *et al.* 2012). Transcriptional interference, as has been observed at the yeast *IME4* gene, presents an alternative mode of regulation by intergenic transcription that does not require alteration of chromatin but can be explained by the collision of traveling Pol II complexes (Prescott and Proudfoot 2002; Hongay *et al.* 2006; Gelfand *et al.* 2011; Hobson *et al.* 2012).

In this study, we sought to expand our knowledge of gene regulatory mechanisms by focusing on genes that harbor noncoding transcription units in their promoters and exploring the importance of both chromatin regulatory proteins and the ncDNA in the regulation of these genes. To begin, we identified candidate genes that might be regulated by noncoding transcription and selected one of these genes, *ECM3*, for detailed study. Our analyses of *ECM3* expression revealed an integral role for the Paf1 complex, H3 K4 methylation, and two histone acetyltransferases in the positive regulation of *ECM3*. In addition, our findings indicate that transcription of the intergenic ncDNA at *ECM3* correlates with expression of the gene, potentially through establishment of a permissive chromatin structure.

## Materials and Methods

### S. cerevisiae strains and media

*S. cerevisiae* strains used in this study are listed in Supplemental Material, Table S1. The strains used to perform the anchor away experiment are W303 derivatives purchased from Euroscarf or generously provided by Patrick Cramer (Schulz *et al.* 2013). For anchor away experiments, cells were grown at 30° in YPD medium (1% yeast extract, 2% peptone, and 2% dextrose) until cultures reached a density of 2 × 10^7^ cells per ml. Rapamycin was then added to cultures for 1 hr at a final concentration of 1 μg/ml from a stock of 1 mg/ml rapamycin suspended in ethanol. All other strains used in this study are derived from a *GAL2^+^* S288C isolate using standard genetic crosses and transformations (Winston *et al.* 1995). The *EUC1* promoter deletions were made by two-step integration of an HA-*URA3*-HA cassette that was PCR-amplified from the plasmid pMPY-3XHA (Schneider *et al.* 1995). This resulted in strains where a portion of the *EUC1* promoter has been replaced with a DNA sequence encoding one copy of the 3XHA tag, which is serving as spacer DNA. Cells were grown at 30° in YPD medium until cultures reached a density of 1–2 × 10^7^ cells per ml for isolation of either RNA or chromatin for use in northern blotting, primer extension, and chromatin immunoprecipitation (ChIP) analyses.

### Northern blot analysis

Northern blot analyses were performed using 20 μg total RNA samples resolved in gels containing 2% agarose, 6.5% formaldehyde, and 1 × MOPS as previously described (Ausubel 1987). Double-stranded probes were generated by random-primed labeling and single-stranded probes were generated by asymmetric PCR with α-^32^P-dATP (Rio 2011). Probe templates were amplified from genomic DNA to contain the following sequences relative to the +1 start codon of the protein-coding gene at each locus: *EUC1* (−541 to −100), *ECM3* (+545 to +976), *ARO2-CUT* (−494 to −49), *ARO8-CUT* (−429 to −28), *CLN3-CUT* (−671 to −321), *FET4-CUT* (−479 to −114), *KNH1-CUT* (−496 to −235), and *SCR1* (−182 to +284). *SCR1* RNA levels serve as an internal loading control. The oligonucleotides used to generate probe templates are listed in Table S2. Images were generated by phosphorimaging and quantified using ImageJ software. For each experiment, the data from at least three biological replicates were averaged.

### Primer extension analysis

Primer extension assays were performed as previously described using 20 μg total RNA samples (Ausubel 1987). Sequencing reactions were performed with Sequenase following the manufacturer’s guidelines (Affymetrix USB) using a purified PCR product as a template. Oligonucleotides were gel-purified and end-labeled with γ-^32^P-ATP and T4 polynucleotide kinase using standard protocols (Ausubel 1987). The *EUC1* and *ECM3* transcription start sites were mapped by primer extension using two different oligonucleotides for each major start site listed in Table S2.

### ChIP

Chromatin was isolated and sheared by sonication using a Misonix 3000 sonicator as previously described (Shirra *et al.* 2005). Immunoprecipitations were performed by incubating sheared chromatin with 5 μl antisera to histone H3 (Tomson *et al.* 2011) or 2.5 μl of antibody to H3 K4me3 (Active Motif, catalog number 39159) at 4° overnight, followed by precipitation using Protein A sepharose beads (GE Healthcare) for 2 hr at 4°. All ChIP results were determined by quantitative PCR amplification of immunoprecipitated DNA compared to input DNA. Real-time PCR reactions were performed using SYBR green reagents (Fermentas) and a Step One Plus instrument (Applied Biosystems). The oligonucleotides used for qPCR amplification are listed in Table S2. Data were analyzed using the Pfaffl relative quantitation method (Pfaffl 2001). H3K4me3 occupancy values were normalized to total H3 occupancy values.

### Data availability

The authors state that all data necessary for confirming the conclusions presented in the article are represented fully within the article.

## Results

### Identification of genes with promoter-localized cryptic unstable transcripts (CUTs)

With the goal of identifying chromatin-mediated gene regulatory mechanisms associated with ncDNA transcription, we selected candidate genes that might be regulated by transcription across their promoter regions. Our selection criteria for candidate genes were intentionally simple to minimize the introduction of bias. The only criterion that we required of a candidate gene was the presence of a noncoding transcript over the promoter of a protein-coding gene in a tandem orientation, resembling the structure of the *SRG1-SER3* locus. Several types of noncoding transcripts have been characterized in *S. cerevisiae*. We focused our studies on cryptic unstable transcripts (CUTs), which are short transcripts that are terminated by the Nrd1-Nab3-Sen1 termination pathway, polyadenylated by the TRAMP complex, and degraded by the nuclear exosome (Arndt and Reines 2015; Porrua and Libri 2015). Enabling their detection, degradation of CUTs can be disrupted by deletion of *TRF4*, which encodes a subunit of the TRAMP complex, or *RRP6*, which encodes a catalytic subunit of the nuclear exosome (Wyers *et al.* 2005; Arigo *et al.* 2006; Davis and Ares 2006). Although we anticipate that regulation by intergenic transcription is not limited to the nature of the intergenic transcript, we limited this study to CUTs, as they are rapidly degraded. Due to the instability of CUTs in wild-type cells, we reasoned that regulatory events associated with CUT expression would likely be due to the act of transcription rather than the ncRNA molecule itself. We selected genes from microarray expression data, which mapped CUTs genome-wide (Neil *et al.* 2009), and confirmed that Pol II occupancy was detected across the promoters of these genes in genome-wide ChIP studies (Steinmetz *et al.* 2006). The genes we selected for initial characterization were *ARO2*, *ARO8*, *CLN3*, *ECM3*, *FET4*, and *KNH1*.

**Figure 1:**
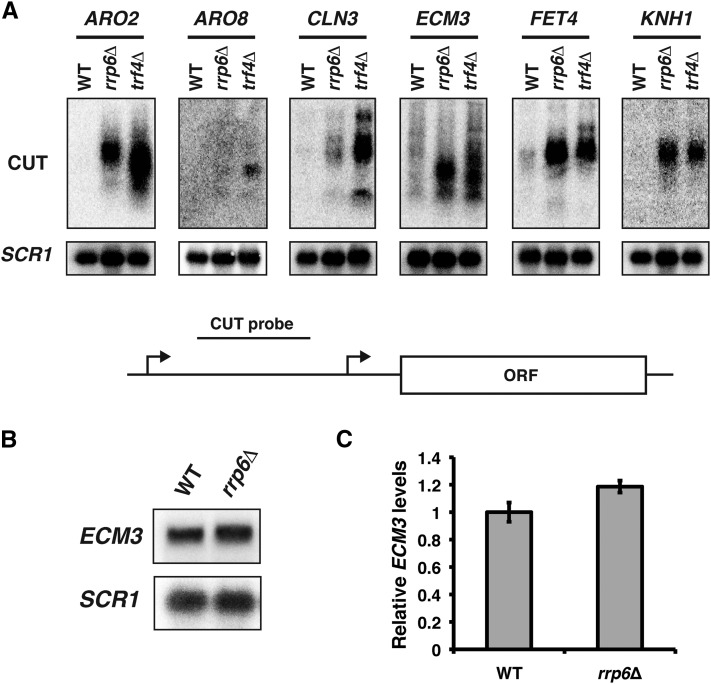
Confirmation of CUT expression at candidate gene promoters. (A) Northern analysis was performed using RNA isolated from a wild-type strain (FY4) or strains where CUTs are stabilized, either *rrp6*Δ (YJ744) or *trf4*Δ (KY1975) mutants. Probes were designed to detect transcripts produced upstream of the neighboring protein-coding gene as diagrammed below. *SCR1* serves as a loading control. (B) Representative northern analysis of *ECM3* mRNA levels in a wild-type strain (YJ1125) compared to an *rrp6*Δ (YJ1126) strain. *SCR1* serves as a loading control. (C) Quantitation of *ECM3* transcript levels in (B) relative to wild-type levels from three biological replicates. Error bars represent the SEM. CUT, cryptic unstable transcript; mRNA, messenger RNA; ORF, open reading frame; WT, wild-type.

To confirm the microarray expression data in our strain background, we performed northern analyses to detect CUT expression over the candidate gene promoters. Using wild-type strains and strains lacking either Rrp6 or Trf4, we were able to detect CUTs over the promoter regions of all six candidate genes (Figure 1A).

We focused our attention on the *ECM3* locus for mechanistic characterization. *ECM3* is a nonessential protein-coding gene that was first identified in a screen for sensitivity to the cell wall stressor, calcofluor white (Lussier *et al.* 1997). For this reason, it is thought that *ECM3* might be involved in cell wall maintenance. We detected two major transcription start sites for *ECM3* (Figure S1), which were also detected by TIF-Seq analysis (Pelechano *et al.* 2013) and which overlap with the 3′ end of the upstream CUT as observed in microarray expression data (Neil *et al.* 2009). This presented an opportunity to observe isoform-specific regulation. We refer to the CUT across the *ECM3* promoter as the *ECM3* upstream CUT (*EUC1*). Upon deletion of the *RRP6* gene, we did not detect a significant change in *ECM3* expression, suggesting that *ECM3* expression is not regulated by stability of the *EUC1* transcript itself (Figure 1, B and C).

### The Paf1 complex and methylation of H3 K4 positively regulate ECM3 expression

**Figure 2:**
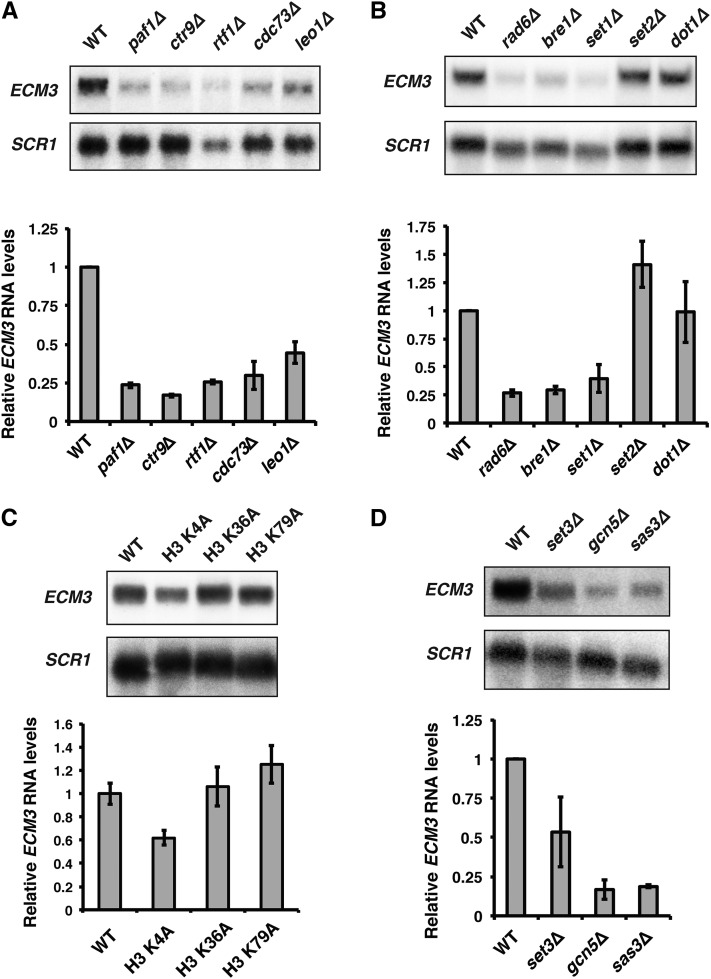
The Paf1 complex and methylation of H3 K4 positively regulate *ECM3* expression. (A) Representative northern blot analysis of *ECM3* transcript levels in a wild-type strain (FY4) or strains lacking one of the five subunits of the Paf1 complex, either *paf1*∆ (YJ807), *ctr9*∆ (KY2170), *rtf1*∆ (YJ788), *cdc73*∆ (KY2171), or *leo1*∆ (KY1805). (B) Representative northern blot analysis of *ECM3* transcript levels in a wild-type strain (FY4) and strains where the genes encoding histone modifiers that work in concert with the Paf1 complex have been deleted (*rad6*∆, KY1712; *bre1*∆, KY1713; *set1*∆, KY2720; *set2*∆, KY2723; and *dot1*∆, KY2725). (C) Representative northern blot analysis of *ECM3* transcript levels in a wild-type control strain, lacking one copy of the genes for H3 and H4 (JDY86), and derivatives of JDY86 in which the only copy of the H3-H4 genes encodes the indicated amino acid substitution in H3. (D) Representative northern blot analysis of *ECM3* transcript levels in strains lacking a subunit of the Set3 HDAC complex (*set3*∆, KY2782), the SAGA HAT complex (*gcn5*∆, KY1743), or the NuA3 HAT complex (*sas3*∆, ECY394) compared to wild-type levels (YJ1125). Quantitation below shows the average *ECM3* mRNA levels relative to WT (set to 1) from at least three biological replicates. Error bars represent the SEM. *SCR1* serves as a loading control. mRNA, messenger RNA; WT, wild-type.

Because noncoding transcription can alter the local chromatin environment in a way that impinges upon protein-coding gene regulation (Fu 2014; Rinn and Guttman 2014; Yang *et al.* 2014), we surveyed various chromatin regulators for their effects on *ECM3* expression. Through these analyses, we determined that the Paf1 complex positively regulates *ECM3* expression, as deletion of any one of the five members of the Paf1 complex results in reduced *ECM3* transcript levels (Figure 2A).

The Paf1 complex associates with Pol II during transcription elongation and plays a crucial role in establishing patterns of histone modifications across transcribed loci (reviewed in Tomson and Arndt 2013). Therefore, to investigate the mechanism by which the Paf1 complex stimulates *ECM3* transcription, we analyzed *ECM3* mRNA levels in strains that lack histone modifiers known to depend on the Paf1 complex for function. Our results indicate that ubiquitylation of H2B K123 and methylation of H3 K4 are required to activate *ECM3* as the enzymes that perform these modifications, the ubiquitin conjugase Rad6 and ubiquitin-protein ligase Bre1 for H2B K123 ubiquitylation and the H3 K4 methyltransferase Set1, are required for normal *ECM3* expression (Figure 2B). Other histone modifications dependent on the Paf1 complex, H3 K79 di- and trimethylation and H3 K36 trimethylation, do not appear to play a role in stimulating *ECM3* transcription. Mutations that delete *DOT1* or *SET2*, which encode the H3 K79 and H3 K36 methyltransferases, respectively, did not reduce *ECM3* mRNA levels (Figure 2B). Targeted mutation of H3 K4 to an unmodifiable residue, H3 K4A, reduced *ECM3* transcript levels, consistent with the idea that Set1 stimulates *ECM3* expression by methylating H3 K4 (Figure 2C). The magnitude of the decrease in *ECM3* mRNA levels was greater for the *set1*∆ mutant than for the H3 K4A mutant. In addition to methylating H3 K4, it is possible that Set1 targets a nonhistone substrate that contributes to *ECM3* expression. Alternatively, because the H3 K4A mutant has only a single copy of the H3 and H4 genes, reduced H3-H4 dosage may partially rescue the H3 K4A effect on *ECM3* transcription. A previous study has shown that histone dosage impacts expression of a gene regulated by noncoding transcription as deletion of a single copy of the H3 and H4 genes, *HHT1* and *HHF1*, results in *SER3* derepression (Hainer and Martens 2011). Furthermore, histone dosage affects the expression of many genes in *S. cerevisiae* (Wyrick *et al.* 1999). Collectively, these data indicate that H2B K123 ubiquitylation by Rad6-Bre1, and subsequent H3 K4 methylation by Set1, positively regulate *ECM3* expression.

Methylation of H3 K4 may serve as a signal to downstream activators of *ECM3* expression. To test this, we explored the effects of complexes that recognize various H3 K4 methylation states on *ECM3* expression. The recruitment of SAGA, NuA3, and NuA4 histone acetyltransferase complexes and the Set3 histone deacetylase complex is stimulated by methylated H3 K4 in *S. cerevisiae* (Martin *et al.* 2006; Ginsburg *et al.* 2009, 2014; Kim and Buratowski 2009; Bian *et al.* 2011). We evaluated the contributions of Set3, SAGA, and NuA3 to *ECM3* regulation by northern analysis using strains lacking a single member of each complex. Consistent with previous studies showing that the Set3 histone deacetylase complex represses transcription (Kim *et al.* 2012), we did not observe a strong or consistent positive effect of *SET3* on *ECM3* expression (Figure 2D). The NuA3 and SAGA complexes have various effects on chromatin and have been generally associated with transcriptional activation. Deletion of the genes encoding Gcn5 and Sas3, the catalytic subunits of SAGA and NuA3, respectively, caused a dramatic reduction in *ECM3* expression (Figure 2D). We note that *ECM3* transcript levels are lower in *gcn5*Δ and *sas3*Δ mutants than in the *set1*Δ mutant. SAGA and NuA3 are both large, multisubunit complexes that associate with chromatin through multiple interactions. It is possible that the loss of H3 K4 methylation results in a partial loss of SAGA and NuA3 activity at *ECM3* and that some function may be retained through other interactions.

### Transcription of the EUC1 ncDNA does not require H3 K4 methylation

**Figure 3:**
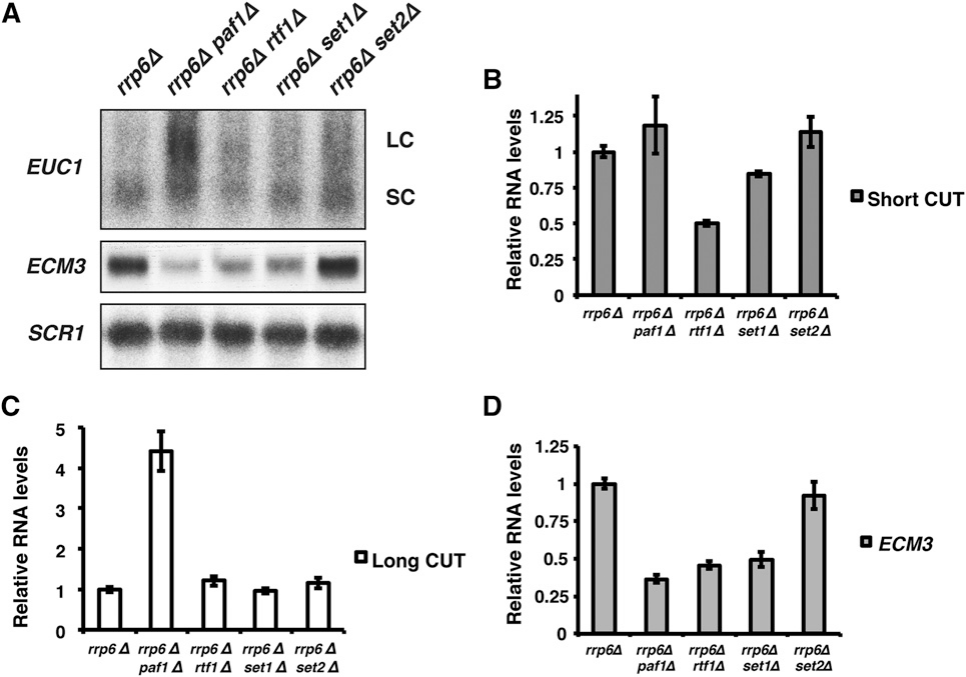
Effect of the Paf1 complex and histone methyltransferases on *EUC1* transcription. (A) Northern blot analysis of RNA isolated from strains lacking *RRP6* to stabilize CUTs and also lacking subunits of the Paf1 complex (*paf1*∆ *rrp6*∆, KY2727 and *rtf1*∆ *rrp6*∆, YJ1143) or histone methyltransferases (*set1*∆ *rrp6*∆, YJ1140 and *set2*∆ *rrp6*∆, YJ1146). The *rrp6*∆ control strain was YJ746. (B–D) Quantitation shows average transcript levels relative to those observed in the *rrp6*∆ strain, which were set to 1. Averaged results from three biological replicates for the *EUC1* short isoform (SC, panel B), the *EUC1* long isoform (LC, panel C), and the *ECM3* ORF transcript (panel D) are shown. Error bars represent the SEM. *SCR1* serves as a loading control. CUT, cryptic unstable transcript; mRNA, messenger RNA; ORF, open reading frame; WT, wild-type.

Expression of many genes is influenced by the regulation of neighboring transcription units. To determine if methylation of H3 K4 could be regulating *ECM3* indirectly by acting farther upstream to regulate transcription of *EUC1*, we analyzed *EUC1* RNA levels in strains lacking H3 K4 methylation. Northern analysis of *EUC1* transcription in an *rrp6*∆ background showed only a slight reduction in *EUC1* transcript levels in the absence of H3 K4 methylation (*rrp6*∆ *set1*∆ strain), although *ECM3* mRNA levels remained low (Figure 3, A and B–D). In the *rtf1*∆ *rrp6*∆ double mutant, *EUC1* levels were lower than those detected in *rrp6*∆ or *set1*∆ *rrp6*∆ strains (Figure 3, A and B–D), consistent with the observation that Rtf1 has functions in addition to regulating H3 K4 methylation (Warner *et al.* 2007). In the absence of Paf1, levels of the *EUC1* isoform present in *rrp6*∆ cells, termed *EUC1* SC for short CUT, were not significantly altered (Figure 3, A and B–D). However, a larger, more abundant *EUC1* isoform, which we refer to as *EUC1* LC for long CUT, was enriched in the *paf1*∆ *rrp6*∆ strain (Figure 3, A and C). Thus, although the *set1*∆, *paf1*∆, and *rtf1*∆ mutations all reduce *ECM3* expression (Figure 3, A and D), they do not affect the pattern of *EUC1* transcripts in the same way. Instead, the correlation with *ECM3* expression is most clearly related to the loss of H3 K4 methylation. Fitting with this idea, a *set2*∆ *rrp6*∆ double mutant, which lacks H3 K36 methylation, does not show a change in *EUC1* or *ECM3* expression compared to the *rrp6*∆ control strain (Figure 3). These data do not exclude a role for *EUC1* in *ECM3* regulation; however, *ECM3* transcript levels remain low in the absence of H3 K4 methylation even though an *EUC1* transcript is produced in the *rrp6*∆ *set1*∆ double mutant strain. Thus, if *EUC1* transcription is involved in *ECM3* regulation, methylation of H3 K4 may work downstream of this effect. These results suggest that H3 K4 methylation does not indirectly regulate *ECM3* expression by acting farther upstream to modulate the levels of *EUC1* transcription.

### Loss of EUC1 transcription correlates with a reduction in ECM3 transcription

**Figure 4:**
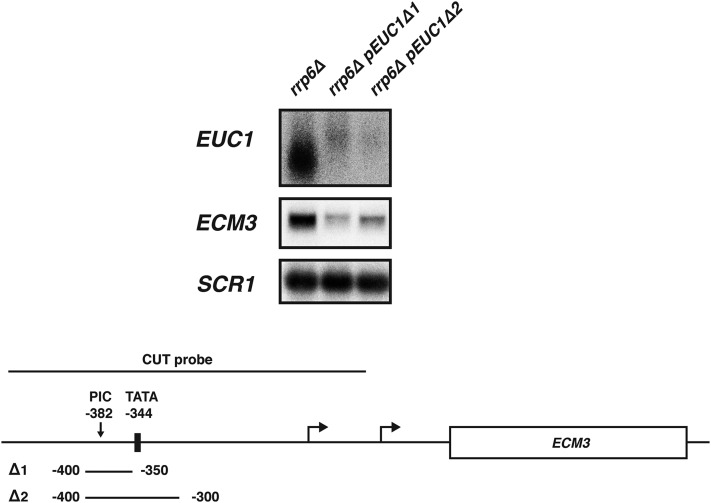
Deletion of putative *EUC1* promoter sequences 5′ of *ECM3* results in reduced *ECM3* expression. Representative northern blot analysis of RNA isolated from strains carrying an *RRP6* deletion and containing either a WT *ECM3* locus (YJ1126) or the indicated *EUC1* promoter deletion mutations. Promoter deletion mutations were introduced at the endogenous *ECM3* locus and replaced either 50 bp (*pEUC1*∆*1*; region −400 to −350 deleted; YJ1128) or 100 bp (*pEUC1*∆*2*; region −400 to −300 deleted; YJ1131) upstream of the +1 start codon of *ECM3*, as diagrammed below. The locations of a PIC identified by Rhee and Pugh (2012) and a putative TATA sequence are indicated on the diagram below. *SCR1* serves as a loading control. CUT, cryptic unstable transcript; PIC, preinitiation complex; WT, wild-type.

As the Paf1 complex associates with actively transcribing polymerases and H3 K4 methylation patterns are established during transcription, one possible model is that transcription of *EUC1* functions to place this modification over the *ECM3* promoter to positively regulate *ECM3* expression. To investigate a possible role for *EUC1* transcription in regulating *ECM3* expression, we devised a strategy to disrupt the *EUC1* promoter and eliminate CUT transcription. To identify putative *EUC1* promoter elements, we performed a sequence alignment of the intergenic region 5′ of *ECM3* in four related yeast species (*S. cerevisiae*, *S. mikatae*, *S. bayanus*, and *S. paradoxus*) (Figure S2). This analysis identified a conserved putative TATA sequence 344 nucleotides upstream of the *ECM3* start codon. In ChIP-exo analyses, Rhee and Pugh (2012) identified a site of preinitiation complex (PIC) assembly 382 nucleotides upstream of the *ECM3* start codon. Guided by this information, we generated promoter deletion mutations at the endogenous *ECM3* locus using a two-step integration method, which replaced the putative *EUC1* promoter sequence with an unrelated DNA sequence (177 bp encoding the 3XHA tag and linker DNA). Deletion 1 (*pEUC1*∆*1*) replaced bases −400–−350 and deletion 2 (*pEUC1*∆*2*) replaced bases −400–−300 relative to the *ECM3* start codon. These deletions eliminate the detected site of PIC assembly and *pEUC1*∆*2* also eliminates the putative TATA sequence (Rhee and Pugh 2012). Both deletions greatly reduce *EUC1* transcript levels (Figure 4). A very small amount of a slightly larger transcript is detected with the *EUC1* probe in RNA isolated from these promoter deletion strains. Based on the location of the northern probes, the nature of the promoter deletion mutations, and the size of the RNA product, this transcript may initiate downstream of the major *EUC1* start sites and extend partially into *ECM3*. Because the amount of this transcript in *pEUC1*∆*2* strains is very small, we continued our analyses with this mutation. Interestingly, both deletions also lower *ECM3* mRNA levels (Figure 4). These results indicate that *EUC1* transcription is positively correlated with *ECM3* transcription.

### Loss of EUC1 transcription is associated with a reduction in H3 K4me3 levels at the ECM3 promoter

**Figure 5:**
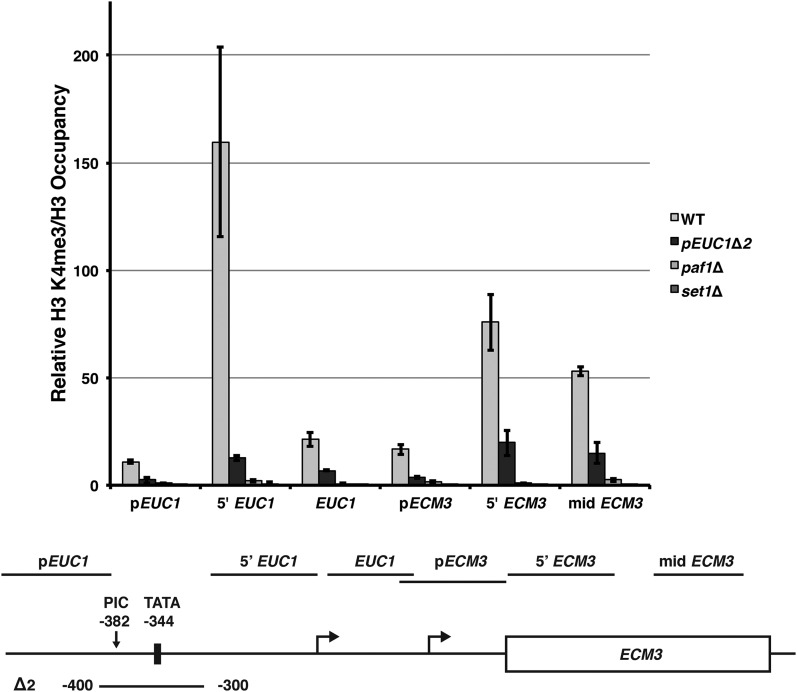
*EUC1* promoter deletion reduces H3K4me3 levels across the *ECM3* locus. ChIP analysis of H3 K4me3 levels at the *ECM3* locus. Immunoprecipitations were performed in biological triplicate using chromatin isolated from WT (FY4, FY5, and YJ1125), *pEUC1*∆*2* (YJ1133, YJ1134, and YJ1135), *paf1*∆ (YJ807, YJ809, and KY1701), and *set1*∆ (KY1755, KY1715, and KY2722) strains. Enrichment of H3 K4me3 relative to input DNA was measured by qPCR and normalized to H3 occupancy. Error bars represent the SEM of three biological replicates. The relative locations of qPCR primers are indicated on the diagram below (the mid *ECM3* primer set is not shown to scale). ChIP, chromatin immunoprecipitation; PIC, preinitiation complex; qPCR, quantitative polymerase chain reaction; WT, wild-type.

To further investigate the connections between H3 K4 methylation and *ECM3* transcription, we analyzed the local chromatin landscape of the *EUC1-ECM3* locus by ChIP analysis. In particular, we analyzed the levels of H3 K4 trimethylation (me3) in the presence and absence of *EUC1* transcription as well as in *paf1*∆ and *set1*∆ mutants, which served as controls for loss of the modification. As expected, H3 K4me3 levels were nearly undetectable in the *paf1*∆ and *set1*∆ mutants (Figure 5). In wild-type cells, two peaks of H3 K4me3 were observed, one over the 5′ end of *EUC1* and one over the 5′ end of *ECM3*. Interestingly, deletion of the putative *EUC1* promoter in the *pEUC1*∆*2* mutant reduced H3 K4me3 levels at both of these locations (Figure 5), consistent with the possibility that *EUC1* transcription positively regulates *ECM3* transcription by promoting H3 K4me3.

### The Paf1 complex and EUC1 impact ECM3 transcription through independent pathways

**Figure 6:**
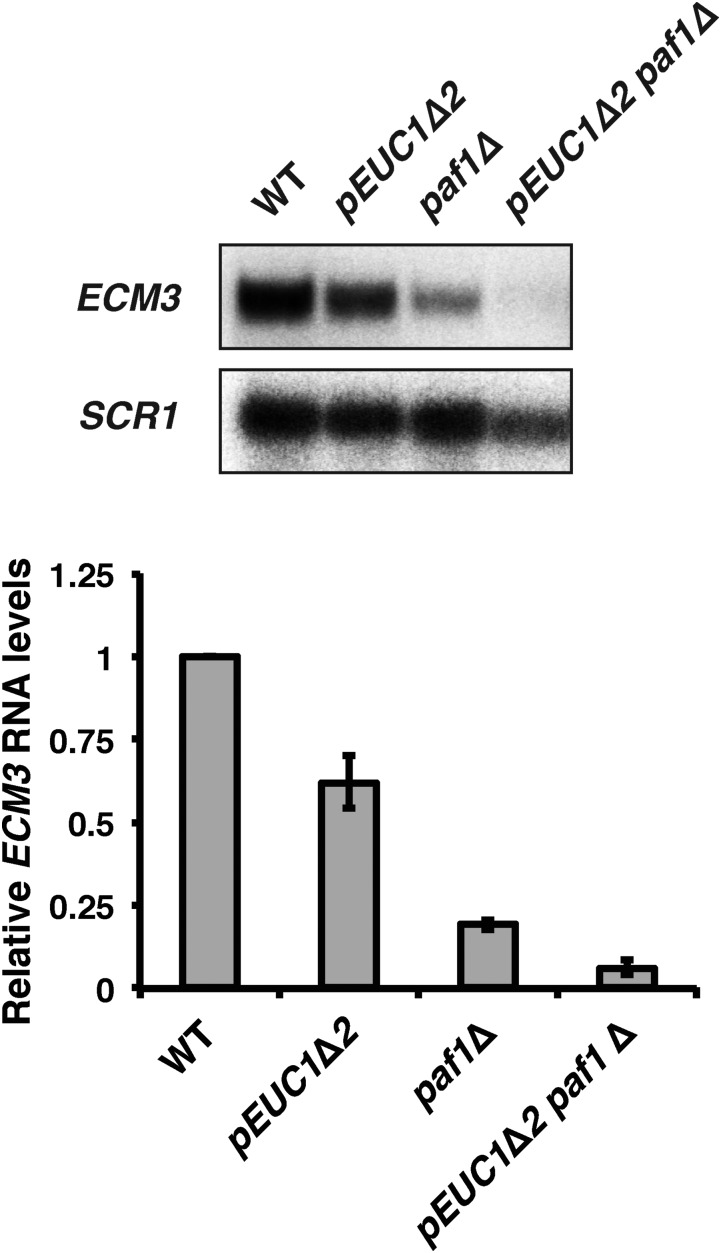
The Paf1 complex and *EUC1* impact *ECM3* expression through independent pathways. Representative northern blot analysis of *ECM3* transcript levels in a *pEUC1*∆*2* strain (YJ1135), a *paf1*∆ strain (KY1701), and a *pEUC1*∆*2 paf1*∆ double mutant strain (YJ1138) compared to a WT strain (YJ1125). Bar graphs show the average *ECM3* mRNA levels relative to WT strains (set to 1) from three biological replicates. Error bars represent the SEM. *SCR1* serves as a loading control. WT, wild-type.

To determine if Paf1 regulates *ECM3* transcription in a manner that requires synthesis of the upstream CUT, we analyzed *ECM3* transcript levels in *pEUC1*∆*2 paf1*∆ double mutants. In combination, deletion of the putative *EUC1* promoter and deletion of *PAF1* caused a greater defect in *ECM3* expression than either mutation alone (Figure 6). Although the interpretation of these data are complicated somewhat by the fact that the *pEUC1*∆*2* mutation is not equivalent to an *EUC1* null allele, as it does not completely eliminate *EUC1* transcription (Figure 4), the results are consistent with the Paf1 complex and *EUC1* transcription having separable roles that contribute to *ECM3* expression. Our data suggest that one shared role of *EUC1* and the Paf1 complex is promoting methylation of H3 K4 (Figure 5), but each of these factors may have other contributions to *ECM3* regulation that remain to be identified.

### Defective termination of EUC1 is not sufficient to repress ECM3 expression

**Figure 7:**
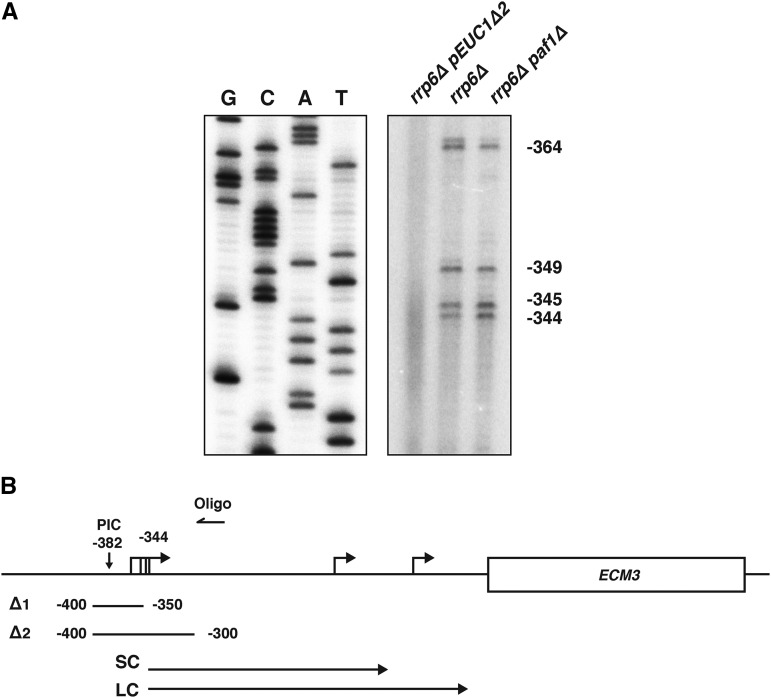
Evidence that the short and long CUT isoforms of *EUC1* initiate from the same transcription start sites. (A) Primer extension analysis of the 5′ ends of *EUC1* transcripts produced in strains that express the *EUC1* SC transcript (*rrp6*∆, YJ746) or both the *EUC1* SC and LC transcripts (*paf1*∆ *rrp6*∆, KY2729). The *pEUC1*∆*2* mutant (YJ1130) was used as a negative control as this strain displays severely reduced *EUC1* transcription. A DNA sequencing ladder is shown on the left. (B) A schematic diagram of the *ECM3* locus with the positions of the upstream *EUC1* CUTs and the *pEUC1*∆*1* and *pEUC1*∆*2* mutations indicated. For simplicity, the *EUC1* SC and LC isoforms are diagrammed as initiating at a single start site to reflect that the closely positioned start sites detected in (A) do not appear as distinct isoforms by northern blot analysis. CUT, cryptic unstable transcript; LC, long CUT; SC, short CUT; PIC, preinitiation complex.

We next investigated whether the long isoform of *EUC1* (LC), which is enriched in *paf1*∆ strains (Figure 3), plays a role in repressing *ECM3* transcription. Northern analysis using strand-specific probes to detect sense transcripts showed that the LC isoform, like the SC isoform, is transcribed from the sense strand relative to the *ECM3* ORF (Figure S3). Neither the SC nor the LC isoform is detected with an antisense strand-specific northern probe. In addition, the LC transcript is not detected in *paf1*∆ strains carrying a wild-type *RRP6* allele, indicating that it is also unstable (Figure S3). Because Paf1 is required for proper termination by the Nrd1-Nab3-Sen1 pathway (Sheldon *et al.* 2005; Tomson *et al.* 2011, 2013), we hypothesized that the longer *EUC1* isoform might be a read-through product of the smaller isoform that terminates farther downstream. In support of this, primer extension analysis revealed that *paf1*∆ *rrp6*∆ strains use the same *EUC1* transcription start sites as an *rrp6*∆ control strain, which are located between 344 and 364 nucleotides upstream of the *ECM3* start codon (Figure 7).

**Figure 8:**
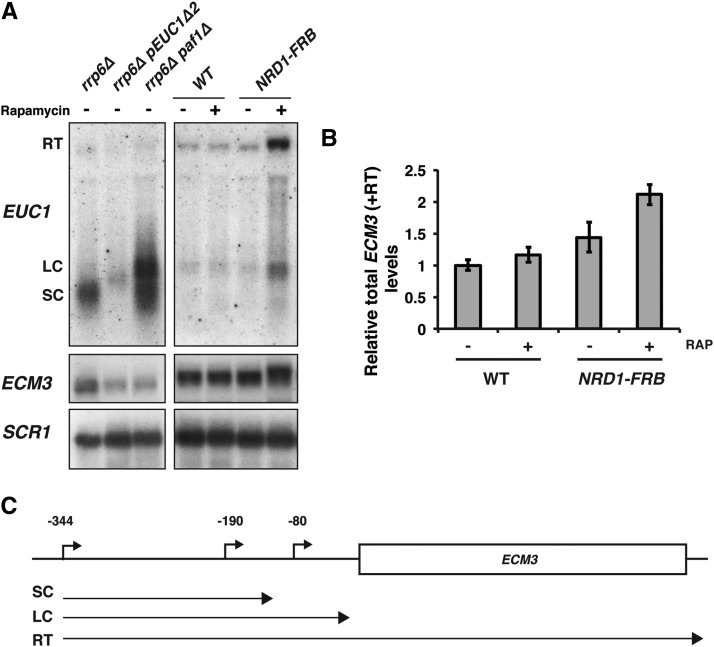
Disruption of CUT termination produces the long *EUC1* isoform. (A) Representative northern blot analysis comparing *EUC1* and *ECM3* transcript patterns in *rrp6*∆ (YJ746), *rrp6*∆ *pEUC1*∆*2* (YJ1131), and *paf1*∆ *rrp6*∆ (KY2729) strains to those of a strain in which Nrd1 (OKA292) has been depleted from the nucleus by the anchor away method. An untagged anchor away strain was used as a control (OKA279). *SCR1* serves as a loading control. The following transcripts were detected with the *EUC1* probe: RT, LC, and SC. Lanes 1–3 and 4–7 were taken from the same exposure of the same blot. Intervening lanes were removed for clarity. (B) Quantitation of *ECM3* levels detected in (A) from three replicates. (+) and (−) indicate the presence or absence of Rap. Quantitation includes signal from the RT isoform and the values are relative to the WT control (−) Rap. Error bars represent the SEM. (C) Diagram showing the relative positions of *EUC1* isoforms at the *ECM3* locus. CUT, cryptic unstable transcript; LC, long CUT; Rap, rapamycin; RT, read through transcript; SC, short CUT; WT, wild-type.

We also analyzed the potential effect of alternative *EUC1* termination by depletion of Nrd1 from the nucleus by the anchor-away method (Haruki *et al.* 2008). Upon depletion of Nrd1 from the nucleus by addition of rapamycin to a *NRD1-FRB* strain, a prominent *EUC1* transcript, which comigrated with the LC isoform observed in the *paf1*∆ background, was observed. This result is consistent with the idea that the LC isoform detected in *paf1*∆ *rrp6*∆ strains arises from transcriptional read-through of a CUT terminator. In the Nrd1-depleted strain, an additional transcript was detected by the *EUC1* probe and this transcript corresponds to the size of an *EUC1* transcript reading through (RT) the entire *ECM3* ORF (Figure 8A). The relative locations of these isoforms based on northern probe hybridization and relative size are diagrammed in Figure 8C. The RT isoform overlaps with the *ECM3* mRNA bands in the northern blot in Figure 8A and is included in quantitation of *ECM3* levels. Upon addition of rapamycin, there is no significant change in *ECM3* transcript levels in the control strain used for the anchor away technique (labeled WT in Figure 8). There is also no significant fold change in *ECM3* transcript levels in the *NRD1-FRB* strain after addition of rapamycin (Figure 8B). This result indicates that a defect in Nrd1-dependent termination of the *EUC1* CUT does not significantly change the overall level of *ECM3* transcripts. Although we have not ruled out a role for the longer *EUC1* isoform in fine-tuning *ECM3* expression, these data indicate that transcription of the long *EUC1* isoform alone is not sufficient to regulate *ECM3* expression under these conditions. Therefore, of the two described functions of the Paf1 complex that we have explored, stimulation of H3 K4 methylation is important for *ECM3* transcription, while regulation of *EUC1* termination plays little if any role in the expression of *ECM3*. As indicated by our genetic data (Figure 6), we anticipate that the Paf1 complex has multiple functions that contribute to *ECM3* regulation that will be interesting to explore.

## Discussion

Prompted by previous mechanistic work that revealed a repressive effect of noncoding transcription on expression of the yeast *SER3* gene, we sought to identify additional cases in which transcription of intergenic DNA upstream of a protein-coding gene impacts the expression of that gene. We focused on the *ECM3* locus, where a promoter-associated CUT, *EUC1*, is synthesized in the sense direction relative to the ORF. We investigated the role of transcription-associated chromatin alterations in the regulation of *ECM3*, as many genes regulated by intergenic transcription employ mechanisms that alter the local chromatin environment. A survey of transcription-associated chromatin regulators uncovered an integral role for the Paf1 complex in the positive regulation of *ECM3* expression. Each of the five subunits of the Paf1 complex is necessary for proper *ECM3* expression. Our data suggest that the role of the Paf1 complex in promoting H3 K4 methylation is required for proper *ECM3* expression. Loss of Set1, the histone methyltransferase for H3 K4, or the ubiquitin conjugase and ligase enzymes that catalyze H2B K123 monoubiquitylation, the prerequisite modification for H3 K4 di- and trimethylation, results in reduced *ECM3* expression. Additionally, we provide evidence that the role of H3 K4 methylation may be to serve as a signal for HAT activity at the *ECM3* promoter. Loss of catalytic subunits of the SAGA and NuA3 HAT complexes, which both recognize methylated states of H3 K4, results in a dramatic reduction of *ECM3* expression. In addition to the stimulatory role of this network of chromatin modifiers, *ECM3* transcription appears to be positively correlated with noncoding transcription across its promoter. Differences in the levels and isoforms of the *EUC1* noncoding transcripts do not strongly affect *ECM3* transcription. Instead, the common link between factors stimulating *ECM3* expression appears to be their role in promoting H3 K4 methylation. Consistent with this idea, a *EUC1* promoter deletion reduced H3 K4me3 levels across both the *EUC1* and *ECM3* transcription units.

One explanation for our observations is that transcription of *EUC1* positively regulates *ECM3* expression by promoting the methylation of H3 K4, which may lead to downstream histone acetylation at the *ECM3* promoter. A key result in support of this model is that deletion of a 50 bp sequence just upstream of the *EUC1* transcription start sites severely impaired both *EUC1* and *ECM3* expression. Although we cannot completely rule out the possibility that this deletion (*pEUC1*∆*1*) may remove a transcription factor binding site that could directly activate the *ECM3* promoter, several pieces of data indicate that this sequence most likely contains key regulatory elements of the *EUC1* promoter. First, our ChIP data show two distinct peaks of H3 K4me3 for the *EUC1* and *ECM3* transcripts, suggesting that these promoters are distinct. Second, preliminary data in environmental conditions where we have observed slight changes in *ECM3* expression show a corresponding change in expression of the short *EUC1* isoform. Although these effects are subtle, they suggest that the correlation between *EUC1* and *ECM3* expression is not limited to a context where the *EUC1* promoter has been deleted. Third, we report multiple lines of evidence for the methylation of H3 K4 as a positive regulatory event for *ECM3* expression. As this histone modification is coupled to transcriptional activity, it is likely that transcription of *EUC1* plays a role in placing this mark across the *ECM3* promoter. Though we are not aware of any transcription factors that both bind to the sequence deleted in *pEUC1*∆*1* and upregulate *ECM3* transcription in a manner not dependent on *EUC1*, we cannot rule out their existence. Our attempts to prevent *EUC1* transcription through strategies other than generating the *pEUC1*∆*1* and *pEUC1*∆*2* mutations were unsuccessful. Introduction of an exogenous terminator sequence within *EUC1* had nonspecific effects on *ECM3* transcription start site selection, and targeted mutation of the predicted TATA element failed to eliminate *EUC1* transcription. This is consistent with previous reports detecting PIC assembly further upstream of the putative TATA sequence and indicates that this TATA element is not likely to be the predominant site of PIC assembly for the *EUC1* transcript. The overall AT-richness of this region may allow for transcription initiation at multiple sites that may not be abolished by targeted mutations.

While both appear to be important for establishing the H3 K4 methylation pattern across the *ECM3* promoter, *EUC1* transcription and the Paf1 complex also have separable functions in facilitating *ECM3* expression. A double mutant lacking both *EUC1* transcription and *PAF1* is more severely compromised for *ECM3* expression than either single mutant strain. This indicates that the Paf1 complex stimulates *ECM3* transcription through a mechanism in addition to its role in promoting H3 K4 methylation. This is further evidenced by the Leo1 subunit of the Paf1 complex having a positive role in *ECM3* regulation, despite the fact that Leo1 is not required for H3 K4me3 (Ng *et al.* 2003). Although termination of the *EUC1* transcript is altered in a *paf1*∆ strain, our results of Nrd1 depletion do not support a major role for alternative termination of *EUC1* in regulating levels of *ECM3* expression. However, it is possible that there are transient isoform-specific effects or that these isoforms have different roles under biologically relevant conditions. An intriguing feature of the locus is that the *EUC1* and *ECM3* transcripts overlap in a region of DNA occupied by a nucleosome (Mavrich *et al.* 2008). It is interesting to speculate that the position and modification state of this nucleosome could be a major determinant in promoting or preventing transcription of *ECM3*. This nucleosome could also be a determinant in which isoforms of *EUC1* and *ECM3* are expressed. It would be interesting to relate the position of this nucleosome with where termination of *EUC1* and initiation of *ECM3* occur.

Our finding of a positive role for H3 K4 methylation at a gene that lies downstream of a noncoding transcription unit is interesting, as others have shown this modification to be repressive to initiation of transcription at the *GAL1-10*, *DCI1*, and *DUR3* loci (Houseley *et al.* 2008; Pinskaya *et al.* 2009; Kim *et al.* 2012). In these cases, noncoding transcription places H3 K4 methylation across the promoters of these genes and results in the recruitment of HDACs, such as Set3. Our data indicate that the functionally important readers of the H3 K4 methyl marks at *ECM3* are the HAT complexes SAGA and NuA3, which would explain the opposite effect of H3 K4 methylation on *ECM3* expression compared to *GAL1-10*, *DCI1*, and *DUR3*. Differential recruitment of readers could depend on whether the histones at the promoters of these genes display predominantly tri- or dimethylation of H3 K4 or other differences in the local chromatin environment. The observation that one histone modification associated with noncoding transcription can have opposite effects on the regulation of a neighboring gene adds to the increasing diversity of mechanisms by which noncoding transcription can regulate gene expression. This points to a potential plethora of regulatory mechanisms imparted by noncoding transcription that will be important to study at individual genes.

## Supplementary Material

Supplemental Material
