# Supplementary material for: Evidence for Regulation of ECM3 Expression by Methylation of Histone H3 Lysine 4 and Intergenic Transcription in Saccharomyces cerevisiae
Source: G3 (Bethesda). 2016 Jul 22;6(9):2971–81. doi: 10.1534/g3.116.033118 (PMC5015954; doi:10.1534/g3.116.033118)
Supplement: Supplemental Material [file supp_6_9_2971__index.html]

Evidence for Regulation of ECM3 Expression by Methylation of Histone H3 Lysine 4 and Intergenic Transcription in Saccharomyces cerevisiae — Supplemental Material 

# Evidence for Regulation of *ECM3* Expression by Methylation of Histone H3 Lysine 4 and Intergenic Transcription in *Saccharomyces cerevisiae*

## Supplemental Material for Raupach, *et al*, 2016

**Files in this Data Supplement:**

- Figure S1 - Identification of two major transcription start sites for *ECM3*. (.pdf, 298 KB)
- Figure S2 - Multiple sequence alignment of the intergenic region upstream of the *ECM3* ORF in four related yeast species. (.pdf, 751 KB)
- Figure S3 - The long *EUC1* isoform is an unstable transcript that is transcribed in the sense direction relative to *ECM3*. (.pdf, 344 KB)
- Table S1 - *Saccharomyces cerevisiae* strains used in this study. (.pdf, 45 KB)
- Table S2 - Oligonucleotides used in this study. (.pdf, 37 KB)
