# Supplementary material for: Evidence for Regulation of ECM3 Expression by Methylation of Histone H3 Lysine 4 and Intergenic Transcription in Saccharomyces cerevisiae
Source: G3 (Bethesda). 2016 Jul 22;6(9):2971–81. doi: 10.1534/g3.116.033118 (PMC5015954; doi:10.1534/g3.116.033118)
Supplement: Supplemental Material [file supp_g3.116.033118_FigureS1.pdf]

FIGURE S1

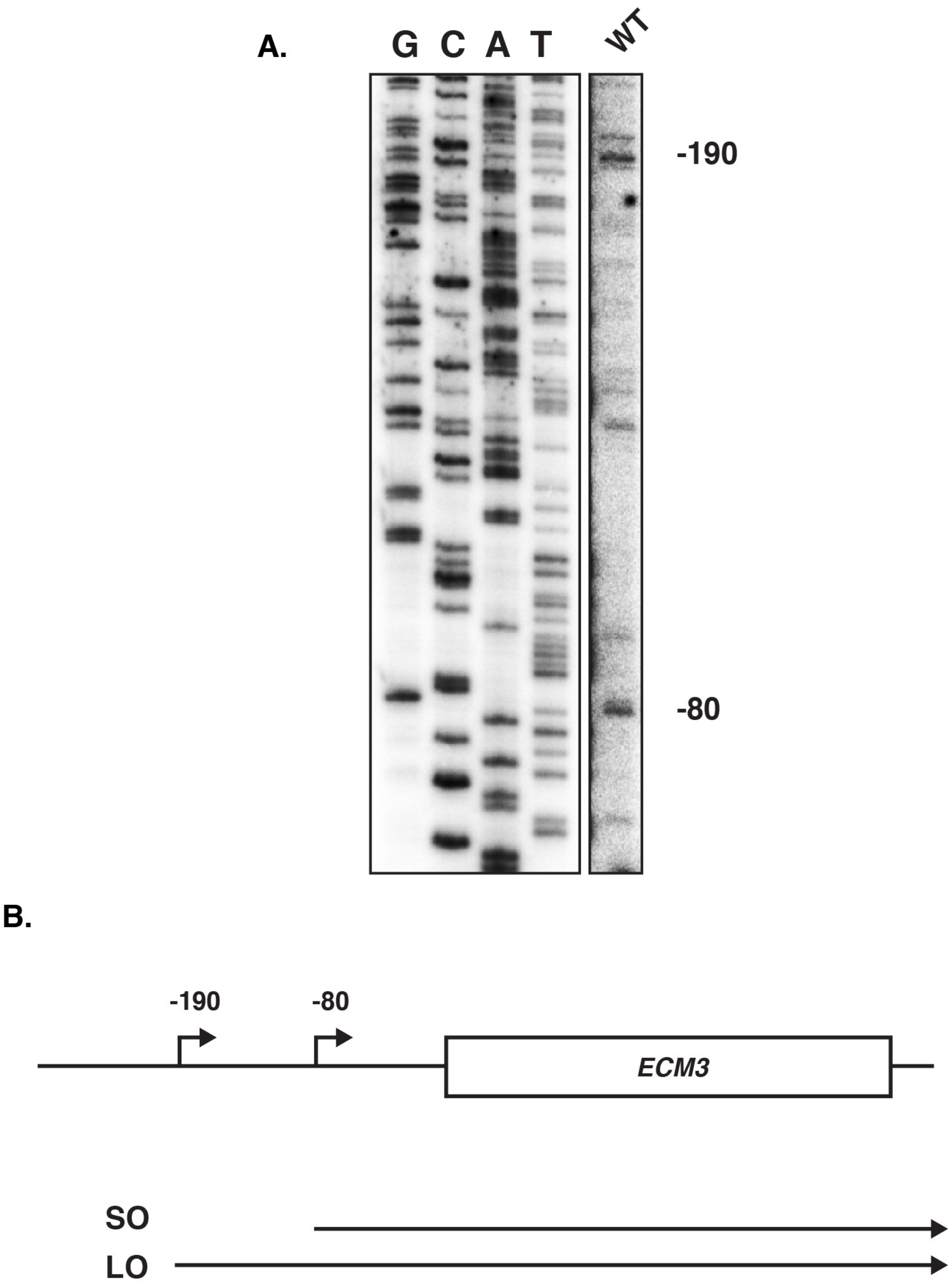

Figure S1. Identification of two major transcription start sites for *ECM3*. (A) Primer extension analysis was performed using RNA isolated from a wild-type strain (FY5). The oligonucleotide used to map these start sites is listed in Table S2. These start sites were identified using two different oligonucleotides and RNA isolated from more than one strain. (B) Diagram of the results shown in (A). *ECM3* shows at least two major start sites initiating 80 bases and 190 bases upstream of the +1 ATG of the *ECM3* ORF. The isoform initiating at -80 is referred to as the short ORF (SO) isoform and the isoform initiating at -190 is referred to as the long ORF (LO) isoform.
