# Supplementary material for: Evidence for Regulation of ECM3 Expression by Methylation of Histone H3 Lysine 4 and Intergenic Transcription in Saccharomyces cerevisiae
Source: G3 (Bethesda). 2016 Jul 22;6(9):2971–81. doi: 10.1534/g3.116.033118 (PMC5015954; doi:10.1534/g3.116.033118)
Supplement: Supplemental Material [file supp_g3.116.033118_FigureS2.pdf]

FIGURE S2

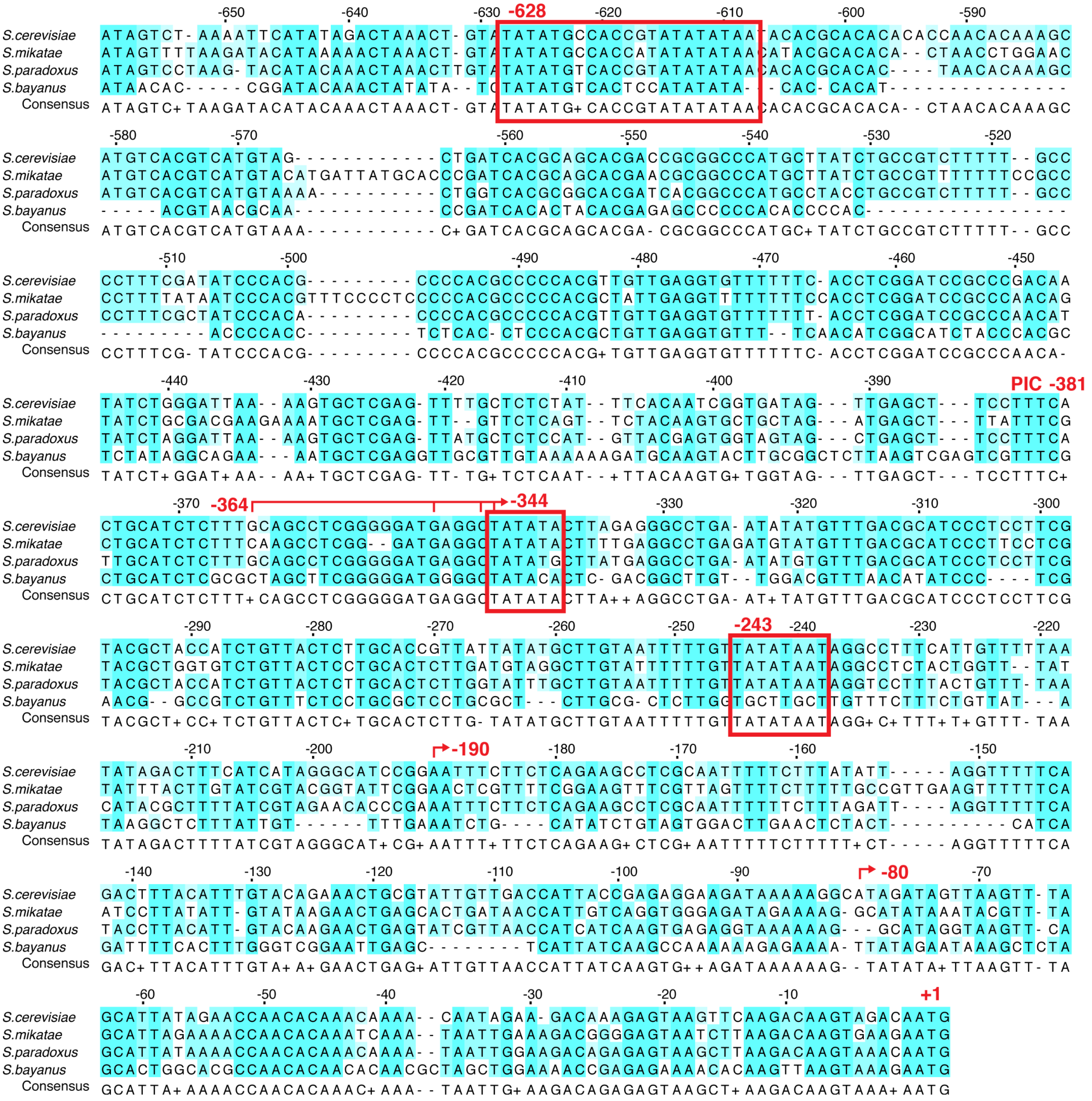

Figure S2. Multiple sequence alignment of the intergenic region upstream of the *ECM3* ORF in four related yeast species. Sequences in this alignment were obtained from the *Saccharomyces cerevisiae* genome database. Alignment of these sequences was performed using Clustal X software and this image was generated using Jalview and Adobe Illustrator software. The alignment was performed including sequence from the upstream tRNA gene extending into the *ECM3* ORF. The first three nucleotides in this image are the 3' end of the upstream tRNA gene. The last three nucleotides in this image are the +1 start codon of the *ECM3* ORF. Darker cyan indicates a higher degree of sequence similarity. The locations of putative TATA sequences are highlighted in red boxes. The location of the PIC identified by Rhee and Pugh (2012) is indicated above the sequence and corresponds to the midpoint of TFIIB occupancy. Transcription start sites mapped by primer extension in Figures S1 and 7 for the *EUC1* and *ECM3* transcripts are marked as red arrows.
