# Supplementary material for: Evidence for Regulation of ECM3 Expression by Methylation of Histone H3 Lysine 4 and Intergenic Transcription in Saccharomyces cerevisiae
Source: G3 (Bethesda). 2016 Jul 22;6(9):2971–81. doi: 10.1534/g3.116.033118 (PMC5015954; doi:10.1534/g3.116.033118)
Supplement: Supplemental Material [file supp_g3.116.033118_FigureS3.pdf]

FIGURE S3

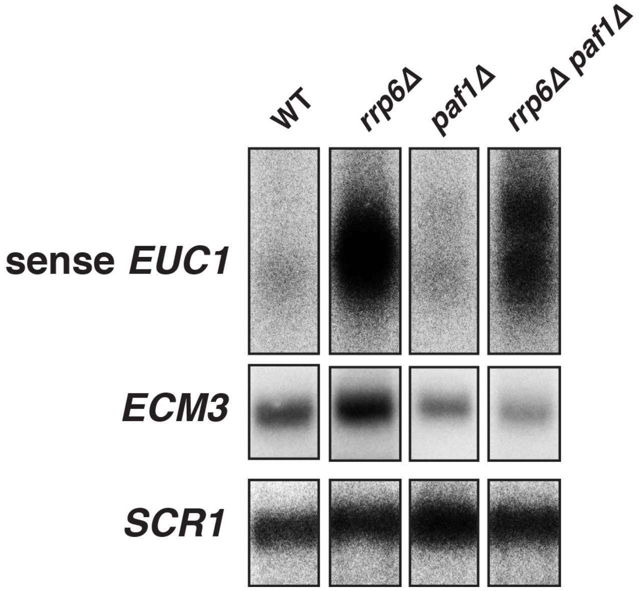

Figure S3. The long *EUC1* isoform is an unstable transcript that is transcribed in the sense direction relative to *ECM3*. Representative northern analysis of *EUC1* and *ECM3* levels in a wild-type strain compared to strains where CUTs are stabilized (*rrp6*Δ) or lacking Paf1 (*paf1*Δ and *paf1*Δ *rrp6*Δ). *EUC1* was detected using a sense strand-specific probe labeled by asymmetric PCR. *SCR1* serves as a loading control.
