# Supplementary material for: Evidence for Regulation of ECM3 Expression by Methylation of Histone H3 Lysine 4 and Intergenic Transcription in Saccharomyces cerevisiae
Source: G3 (Bethesda). 2016 Jul 22;6(9):2971–81. doi: 10.1534/g3.116.033118 (PMC5015954; doi:10.1534/g3.116.033118)
Supplement: Supplemental Material [file supp_g3.116.033118_TableS1.pdf]

Table S1. ***Saccharomyces cerevisiae* strains used in this study**.

Strain	Genotype	Reference or Source
FY4	*MATa*	Winston, *et al*., 1995
FY5	*MATα*	Winston, *et al*., 1995
JDY86	*MATa his3Δ200 leu2Δ0 lys2Δ0 trp1Δ63 ura3Δ0 met15Δ0 can1::MFA1pr-HIS3 hht1-hhf1::*$_{NatMX4}$ *hht2-hhf2::[HHTS-HHFS]-URA3*	Dai *et al*., 2008
JDY86 derivative	*MATa his3Δ200 leu2Δ0 lys2Δ0 trp1Δ63 ura3Δ0 met15Δ0 can1::MFA1pr-HIS3 hht1-hhf1::NatMX4 hht2-hhf2::[hhts-K4A-HHFS]-URA3*	Dai *et al*., 2008
JDY86 derivative	*MATa his3Δ200 leu2Δ0 lys2Δ0 trp1Δ63 ura3Δ0 met15Δ0 can1::MFA1pr-HIS3 hht1-hhf1::NatMX4 hht2-hhf2::[hhts-K36A-HHFS]-URA3*	Dai *et al*., 2008
JDY86 derivative	*MATa his3Δ200 leu2Δ0 lys2Δ0 trp1Δ63 ura3Δ0 met15Δ0 can1::MFA1pr-HIS3 hht1-hhf1::NatMX4 hht2-hhf2::[hhts-K79A-HHFS]-URA3*	Dai *et al*., 2008
KY930	*MATα ura3-52 lys2-128δ rad6Δ0::URA3*	M. Braun and K. Arndt, unpublished
KY1130	*MATα his3Δ200 leu2Δ1 bre1Δ0::KanMX*	M. Braun and K. Arndt, unpublished
KY1700	*MATα paf1Δ0::KanMX*	Crisucci and Arndt, 2011
KY1701	*MATa leu2Δ0 paf1Δ0::KanMX*	Crisucci and Arndt, 2012
KY1703	*MATa rtf1Δ0::KanMX*	Crisucci and Arndt, 2011
KY1704	*MATα rtf1Δ0::KanMX*	Crisucci and Arndt, 2011
KY1705	*MATa ctr9Δ0::KanMX*	Crisucci and Arndt, 2011
KY1706	*MATα cdc73Δ0::KanMX*	Crisucci and Arndt, 2011
KY1711	*MATa rad6Δ0::KanMX*	Crisucci and Arndt, 2011
KY1712	*MATα rad6Δ0::KanMX*	E. Crisucci and K. Arndt, unpublished
KY1713	*MATα bre1Δ0::KanMX*	Crisucci and Arndt, 2011
KY1715	*MATa set1Δ0::KanMX*	Crisucci and Arndt, 2011
KY1716	*MATa set2Δ0::KanMX*	Crisucci and Arndt, 2011
KY1717	*MATa dot1Δ0::KanMX*	Crisucci and Arndt, 2011
KY1743	*MATα gcn5Δ0::NatMX*	E. Crisucci and K. Arndt, unpublished
KY1755	*MATα set1Δ0::KanMX*	Crisucci and Arndt, 2011
KY1805	*MATα leo1Δ0::KanMX*	Tomson *et al*., 2013
KY1975	*MATa trf4Δ0::NatMX leu2Δ0 ura3Δ0*	E. Crisucci and K. Arndt, unpublished
KY2170	*MATa leu2Δ1 ctr9Δ0::KanMX*	E. Crisucci and K. Arndt, Pruneski *et al*., 2011
KY2171	*MATa cdc73Δ0::KanMX*	E. Crisucci and K. Arndt, unpublished
KY2173	*MATa bre1Δ0::KanMX*	E. Crisucci and K. Arndt, unpublished
KY2377	*MATα rrp6Δ0::kanMX paf1Δ0::kanMX*	Tomson *et al*., 2013
KY2720	*MATa leu2Δ0 set1Δ0::KanMX*	E. Crisucci and K. Arndt, unpublished

KY2721	*MATα ura3Δ0 set1Δ0::KanMX*	E. Crisucci and K. Arndt, unpublished
KY2722	*MATα set1Δ0::KanMX*	E. Crisucci and K. Arndt, unpublished
KY2723	*MATα ura3Δ0 set2Δ0::KanMX*	E. Crisucci and K. Arndt, unpublished
KY2724	*MATa leu2Δ0 set2Δ0::KanMX*	E. Crisucci and K. Arndt, unpublished
KY2725	*MATa leu2Δ0 dot1Δ0::KanMX*	E. Crisucci and K. Arndt, unpublished
KY2726	*MATα ura3Δ0 dot1Δ0::KanMX*	E. Crisucci and K. Arndt, unpublished
KY2727	*MATα rrp6Δ0::kanMX paf1Δ0::kanMX*	E. Crisucci and K. Arndt, unpublished
KY2728	*MATα rrp6Δ0::kanMX paf1Δ0::kanMX*	E. Crisucci and K. Arndt, unpublished
KY2729	*MATα rrp6Δ0::kanMX paf1Δ0::kanMX leu2Δ0*	E. Crisucci and K. Arndt, unpublished
KY2782	*MATα leu2Δ0 set3Δ0::NatMX*	E. Crisucci and K. Arndt, unpublished
KY2783	*MATa leu2Δ0 ura3-52 sas3Δ0::NatMX*	E. Crisucci and K. Arndt, unpublished
OKA279	*MATα tor1-1 frp1::NAT RPL13A-2XFKBP12::TRP1 his3-11,15 leu2-3,112 ura3 trp1-1 ade2-1 can1-100 GAL psi$^+$*	Euroscarf
OKA292	*MATα tor1-1 frp1::NAT RPL13A-2XFKBP12::TRP1 his3-11,15 leu2-3,112 ura3 trp1-1 ade2-1 can1-100 GAL psi$^+$ NRD1-FRB::KanMX6*	Shulz *et al.*, 2013
YJ744	*MATa rrp6Δ0::KanMX*	J. Pruneski and J. Martens, Tomson *et al.*, 2013
YJ746	*MATα rrp6Δ0::KanMX*	J. Pruneski and J. Martens, Tomson *et al.*, 2013
YJ760	*MATα ura3Δ0 ctr9Δ0::KanMX*	K. Petrov and J. Martens, unpublished
YJ761	*MATα ura3Δ0 leo1Δ0::KanMX*	K. Petrov and J. Martens, unpublished
YJ764	*MATa lys2Δ0 ura3Δ0 his3Δ200 cdc73Δ0::KanMX*	K. Petrov and J. Martens, unpublished
YJ766	*MATa lys2Δ0 ura3Δ0 his3Δ200 leu2Δ0 leo1Δ0::KanMX*	K. Petrov and J. Martens, unpublished
YJ788	*MATα his3Δ200 rtf1Δ0::KanMX*	K. Petrov and J. Martens, unpublished
YJ807	*MATα his3Δ200 paf1Δ0::KanMX*	K. Petrov and J. Martens, unpublished

YJ809	*MATα ura3Δ0 paf1Δ0::KanMX*	K. Petrov and J. Martens, unpublished
YJ1125	*MATa ura3Δ0 his3Δ200 leu2Δ0*	This study
YJ1126	*MATa rrp6Δ0::KanMX ura3Δ0 his3Δ200 leu2Δ0*	This study
YJ1127	*MATα rrp6Δ0::KanMX ura3Δ0 his3Δ200 leu2Δ0 ecm3-pEUC1Δ1::HA (-400 to -350)*	This study
YJ1128	*MATa rrp6Δ0::KanMX ura3Δ0 ecm3-pEUC1Δ1::HA (-400 to -350)*	This study
YJ1129	*MATα rrp6Δ0::KanMX ura3Δ0 ecm3-pEUC1Δ1::HA (-400 to -350)*	This study
YJ1130	*MATα rrp6Δ0::KanMX ura3Δ0 leu2Δ0 ecm3-pEUC1Δ2::HA (-400 to -300)*	This study
YJ1131	*MATa rrp6Δ0::KanMX ura3Δ0 his3Δ200 ecm3-pEUC1Δ2::HA (-400 to -300)*	This study
YJ1132	*MATα rrp6Δ0::KanMX ura3Δ0 leu2Δ0 ecm3-pEUC1Δ2::HA (-400 to -300)*	This study
YJ1133	*MATa ura3Δ0 ecm3-pEUC1Δ2::HA (-400 to -300)*	This study
YJ1134	*MATα ecm3-pEUC1Δ2::HA (-400 to -300)*	This study
YJ1135	*MATa leu2Δ0 ecm3-pEUC1Δ2::HA (-400 to -300)*	This study
YJ1136	*MATa paf1Δ0::kanMX his3Δ200 ecm3-pEUC1Δ2::HA (-400 to -300)*	This study
YJ1137	*MATα paf1Δ0::kanMX ura3Δ0 his3Δ200 ecm3-pEUC1Δ2::HA (-400 to -300)*	This study
YJ1138	*MATα paf1Δ0::kanMX ura3Δ0 his3Δ200 ecm3-pEUC1Δ2::HA (-400 to -300)*	This study
YJ1139	*MATα rrp6Δ0::kanMX set1Δ0::kanMX his3Δ200*	This study
YJ1140	*MATa rrp6Δ0::kanMX set1Δ0::kanMX*	This study
YJ1141	*MATa rrp6Δ0::kanMX set1Δ0::kanMX his3Δ200*	This study
YJ1142	*MATa rrp6Δ0::kanMX rtf1Δ0::kanMX*	This study
YJ1143	*MATα rrp6Δ0::kanMX rtf1Δ0::kanMX ura3Δ0 his3Δ200*	This study
YJ1144	*MATa rrp6Δ0::kanMX rtf1Δ0::kanMX leu2Δ0*	This study
YJ1145	*MATα rrp6Δ0::kanMX set2Δ0::kanMX leu2Δ0*	This study
YJ1146	*MATα rrp6Δ0::kanMX set2Δ0::kanMX*	This study
YJ1147	*MATα rrp6Δ0::kanMX set2Δ0::kanMX leu2Δ0 his3Δ200*	This study
