# Supplementary material for: Evidence for Regulation of ECM3 Expression by Methylation of Histone H3 Lysine 4 and Intergenic Transcription in Saccharomyces cerevisiae
Source: G3 (Bethesda). 2016 Jul 22;6(9):2971–81. doi: 10.1534/g3.116.033118 (PMC5015954; doi:10.1534/g3.116.033118)
Supplement: Supplemental Material [file supp_g3.116.033118_TableS2.pdf]

Table S2. **Oligonucleotides used in this study**.

Purpose	Primer set	Oligo name	Orientation	Location relative to +1 ATG	Sequence
**ChIP qPCR**	**p*EUC1***	OJ1172	F	-541	5’-CCATGCTTATCTGCCGTCTT
		OJ1257	R	-381	5’-AGGAAGCTCAACTATCACCG
**ChIP qPCR**	**5’ *EUC1***	OJ1730	F	-299	5’-CGTACGCTACCATCTGTTACTCTTGC
		OJ1731	R	-191	5’-CCGGATGCCCTATGATGAAAGTCTATAT
**ChIP qPCR**	***EUC*1**	OJ1329	F	-186	5’-CTTCTCAGAAGCCTCGCAAT
		OJ1173	R	-100	5’-GGTAATGGTCAACAATACGC
**ChIP qPCR**	**p*ECM3***	OJ1715	F	-119	5’-GCGTATTGTTGACCATTACC
		OJ1270	R	-1	5’-TGTCTACTTGTCTTGAACTTAC
**ChIP qPCR**	**5’ *ECM3***	OJ1732	F	+3	5’-ACACACATCACACTGGGACAAG
		OJ1733	R	+142	5’-CGATATCAGAGATGGACCTTGTG
**ChIP qPCR**	**mid *ECM3***	OJ1728	F	+838	5’-AGCGAGCTTAACGATCCTACT
		OJ1175	R	+976	5’-GTAGTTCACGCATATCGATGG
**Northern probe template**	***ARO2* CUT**	OJ1160	F	-494	5’- CTACGTTGGGCACGTCTAAG
		OJ1161	R	-49	5’- ATAACGCTTAGATGATGCCGT
**Northern probe template**	***ARO8* CUT**	OJ1156	F	-429	5’- CATGGCTCATATACACCATCC
		OJ1157	R	-28	5’- TGTCTGTATCAACTGCAGGG
**Northern probe template**	***CLN3* CUT**	OJ1164	F	-671	5’- GGAAGTGTCGCAACCAAACG
		OJ1166	R	-321	5’- GGCAGACTCAGTAGTAGAAG
**Northern probe template**	***FET4* CUT**	OJ1152	F	-497	5’- GCGTAAATCACACAGGTGTTG
		OJ1153	R	-114	5’- CAATTAATTCATGCCGTGTGAAG
**Northern probe template**	***KNH1* CUT**	OJ1168	F	-496	5’- TCAGCTGTACAAGCCTAGGC
		OJ1169	R	-235	5’- CTGTTGGAGTTGGTCAACAAT
**Northern probe template**	***EUC1***	OJ1172	F	-541	5’- CCATGCTTATCTGCCGTCTT
		OJ1173	R	-100	5’-GGTAATGGTCAACAATACGC
**Northern probe template**	***ECM3***	OJ1174	F	+545	5’- TGACCAATGATGATTCTGCCC
		OJ1175	R	+976	5’- GTAGTTCACGCATATCGATGG
**Northern probe template**	***SCR1***	OJ459	F	-182	5’-CAACTTAGCCAGGACATCCA
		OJ460	R	+284	5’-AGAGAGACGGATTCCTCACG

**Northern probe template**	***ACT1***	OJ257	F	+277	5’-ATCGATTGCTTCATTCTTTTTGTT
		OJ258	R	+845	5’-ATCGATTCTCAAAATGGCGTGAGG
***EUC1* primer extension**		OJ1258	R	-180	5’- GTAACAGATGGTAGCGTACG
***EUC1* primer extension**		OJ1521	R	-192	5’- CGGATGCCCTATGATGAAAGTC
***ECM3* primer extension**		OJ1173	R	-100	5’-GGTAATGGTCAACAATACGC
***ECM3* primer extension**		OJ1270	R	-1	5’-TGTCTACTTGTCTTGAACTTAC
***ECM3* primer extension**		OJ1362	R	+1	5’- CCCAGTGTGATGTGTGTCAT
